# A referral aid for smoking cessation interventions in primary care: study protocol for a randomized controlled trial

**DOI:** 10.1017/S1463423621000244

**Published:** 2021-05-26

**Authors:** Daniëlle N. Zijlstra, Jean W.M. Muris, Catherine Bolman, J. Mathis Elling, Vera E.R.A. Knapen, Hein de Vries

**Affiliations:** 1 Department of Health Promotion, Maastricht University/CAPHRI, Maastricht, the Netherlands; 2 Department of Psychology, Open University of the Netherlands, Heerlen, the Netherlands; 3 Department of General Practice, Maastricht University/CAPHRI, Maastricht, the Netherlands

**Keywords:** evidence-based interventions, general practice, nurse practitioners, primary care, smoking cessation

## Abstract

**Background:**

To expedite the use of evidence-based smoking cessation interventions (EBSCIs) in primary care and to thereby increase the number of successful quit attempts, a referral aid was developed. This aid aims to optimize the referral to and use of EBSCIs in primary care and to increase adherence to Dutch guidelines for smoking cessation.

**Methods:**

Practice nurses (PNs) will be randomly allocated to an experimental condition or control condition, and will then recruit smoking patients who show a willingness to quit smoking within six months. PNs allocated to the experimental condition will provide smoking cessation guidance in accordance with the referral aid. Patients from both conditions will receive questionnaires at baseline and after six months. Cessation effectiveness will be tested via multilevel logistic regression analyses. Multiple imputations as well as intention to treat analysis will be performed. Intervention appreciation and level of informed decision-making will be compared using analysis of (co)variance. Predictors for appreciation and informed decision-making will be assessed using multiple linear regression analysis and/or structural equation modeling. Finally, a cost-effectiveness study will be conducted.

**Discussion:**

This paper describes the study design for the development and evaluation of an information and decision tool to support PNs in their guidance of smoking patients and their referral to EBSCIs. The study aims to provide insight into the (cost) effectiveness of an intervention aimed at expediting the use of EBSCIs in primary care.

## Introduction

Smoking remains the highest contributor to substance-attributable mortality (Peacock *et al.*, 2018). In the Netherlands, around 20,000 people die from firsthand or secondhand smoke inhalation each year (CBS, 2018). Consequently, in 2018, the Dutch government created the National Prevention Act which, among other things, aims to create a smoke-free generation in 2040 (Nationaal Preventieakkoord, 2018). In 2018, 36.9% of Dutch smokers attempted to quit (Bommele and Willemsen, 2019). However, only 5% of those who quit remain abstinent after 12 months (Fiore *et al.*, 2000; Hughes *et al.*, 2004).

The use of an evidence-based smoking cessation intervention (EBSCI) can double the chance of successful smoking cessation after 12 months (West *et al.*, 2015). EBSCIs come in two forms: behavioral counseling and supplementations. Behavioral counseling can consist of face-to-face counseling by a healthcare professional (HCP) such as a general practitioner (GP) or a practice nurse (PN) or trained stop coach outside the GP setting (Pieterse *et al.*, 2001; Aveyard *et al.*, 2012; Stead *et al.*, 2013; Verbiest *et al.*, 2013; Papadakis *et al.*, 2016; van Rossem *et al.*, 2017), tailored online counseling (eHealth) (Te Poel *et al.*, 2009; Stanczyk *et al.*, 2014), telephone counseling (Berndt *et al.*, 2016), and group counseling (McEwen *et al.*, 2006). However, only 25%–30% of smokers report using behavioral counseling methods (Borland *et al.*, 2012; Filippidis *et al.*, 2019). The effectiveness of behavioral counseling can be increased through supplementation with nicotine replacement therapy such as nicotine gum or patches (available over the counter at pharmacies, drugstores, and large supermarkets) (Stead *et al.*, 2012) or pharmacotherapy (Hurt *et al.*, 1997; Hughes *et al.*, 2005; Tonstad *et al.*, 2006) of which the latter can only be prescribed in the Netherlands via the GP setting to patients indicating that they are willing to make use of them (Chavannes *et al.*, 2017).

The general practice setting is a gateway to reach and advise smokers; most Dutch smokers visit their GP yearly (Boerdam and Bevolkingstrends, 2016) and smokers have a high level of trust in their GP (Guassora and Gannik, 2010). Theoretically, a good fit between the treatment and the patient’s needs and preferences improves the patient’s chances of successfully quitting smoking. Discussing intervention options, their characteristics and a subsequent referral to cost-effective EBSCIs, such as eHealth interventions (Cheung *et al.*, 2017; 2018) can assist smokers in finding the best way to quit in accordance with their needs, while also lowering the time burden of smoking cessation counseling during (chronic care) consultations.

The Dutch smoking cessation guidelines for general practices instruct HCPs to actively offer smokers cessation treatment and to refer smokers to EBSCIs that fit patients’ needs and preferences (Chavannes *et al.*, 2017). Up till 10 years ago, smoking cessation support was predominantly provided by the GP. Nowadays, in most Dutch practices, smoking patients are referred to a PN (Dierick-van Daele *et al.*, 2009). PNs are specialized in chronic care (Heiligers *et al.*, 2012), which mostly consists of lifestyle change guidance. GPs and PNs usually use an evidence-based health counseling protocol similar to the 5-A’s (Ask, Advise, Assess, Assist, and Arrange) Tobacco Cessation Guideline (Fiore *et al.*, 2000) as also prescribed by the Dutch smoking cessation guidelines (Chavannes *et al.*, 2017) and previous studies in the GP setting (Pieterse *et al.*, 2001; de Ruijter *et al.*, 2016).

However, PNs’ adherence to smoking cessation guidelines and the referral of patients to fitting EBSCIs is sub-optimal (Cabana *et al.*, 1999; de Ruijter *et al.*, 2017). This may result from PNs unfamiliarity with EBSCIs, which hinders them in confidently discussing options with patients (de Ruijter *et al.*, 2017). Other barriers may be a high workload, a shortage of resources, or an unfavorable perception of the usability of cessation guidelines (Abrahamson *et al.*, 2012; de Ruijter *et al.*, 2017).

In order to expedite the use of EBSCIs in primary care and to thereby increase the number of successful quit attempts, a referral aid was developed. This aid aims to optimize the referral to and use of EBSCIs in primary care and to increase adherence to Dutch guidelines for smoking cessation. This paper aims to describe the development of the referral aid, as well as the design of the associated effectiveness and cost-effectiveness studies.

## Method

### Ethical approval

The medical ethics committee of the University Hospital Maastricht and Maastricht University evaluated the research proposal and indicated that no medical ethical clearance for this study was needed according to the rules of the Medical Research Involving Human Subjects Act (WMO – 2018-1038). The study was registered at the Netherlands Trial Register (NL7020, https://www.trialregister.nl/trial/7020).

### Study design

The aim is to conduct a multi-site two-group parallel randomized controlled trial involving an experimental condition and a control condition. Patients in the control condition will receive care as usual, which usually includes at least a mandatory brief smoking cessation advice and can be supplemented with counseling based on an evidence-based health counseling protocol similar to 5-A’s(Ask, Advise, Assess, Assist, and Arrange) Tobacco Cessation Guideline (Fiore *et al.*, 2000) as also described in the Dutch guidelines for smoking cessation (Chavannes *et al.*, 2017) and previous studies in the GP setting (Pieterse *et al.*, 2001; de Ruijter *et al.*, 2016) in the way the individual PN sees fit. In addition to the usual care, patients in the experimental condition will receive guidance and referral by the PN in accordance with the referral aid in order to select an EBSCI that fits their needs and preferences, which acts as an expansion on the Assist and Arrange steps from the 5-A’s protocol (Fiore *et al.*, 2000). The chosen EBSCI can either be administered by the PN (i.e., face-to-face counseling) or coordinated by the PN (e.g., eHealth, telephone counseling).

Randomization will occur on a practice level to prevent bias between the PN level or the patients level. General practices will be randomly allocated to either the control condition or the experimental condition in a 1:1 ratio. Randomization will take place via a random number generator that creates a string of numbers (1 = control condition, 2 = experimental condition), which will be allocated to general practices in order of registration. Patients are allocated based on their GP practice allocation. By allocation on the practice level, PNs cannot accidentally bias patients from the control group with information intended for the experimental group. As PNs from the experimental condition are provided with an intervention and PNs from the control condition are only asked to provide care as usual, blinding of the PNs is impossible. Patients will be semi-blinded, as they are unaware of the procedure of any other group than the one they attend.

**Figure 1.**
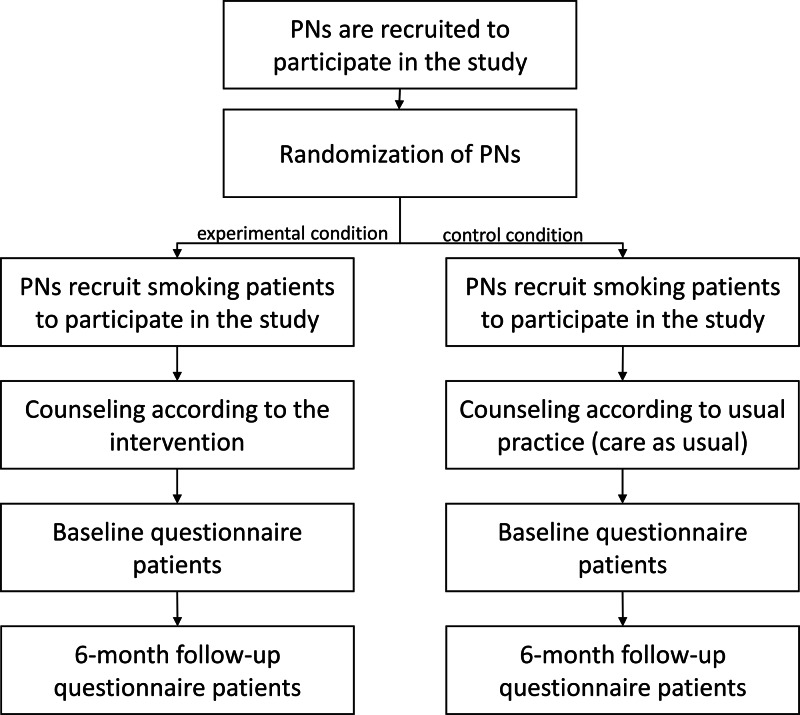
Flowchart of the study design

Patients have to answer two questionnaires – at baseline and follow-up. This can be done on paper or online. Patients receive a link to the questionnaire or a paper version after they have been registered by the PN. The follow-up measurement will take place six months after the baseline questionnaire has been answered. The study design is illustrated in Figure 1.

### Recruitment of practice nurses

From January 2019 until May 2020, PNs in the Netherlands will be approached to participate in the study. The task of the PNs will be twofold – recruiting smokers and referral to EBSCIs in accordance with the intervention’s method.

An information package including a study invitation letter and an intervention summary will be sent to general practices spread over the Netherlands. Dutch primary care associations who support the study will also distribute the information letters to their associate general practices through their own communication channels. To gauge the interest in participating in the study, approached practices will be contacted via telephone after two weeks. HCPs expressing an interest in participating will be sent a more detailed guideline for the study and will be asked to sign a study participation form. PNs are requested to recruit 10–20 patients each. To prevent attrition and stimulate active recruitment by the PNs, participating PNs who recruit at least five patients will receive a remuneration of €100. Inclusion criteria will be that PNs are employed by one or more general practices in the Netherlands and they indicate that they provide smoking cessation counseling as such.

### Recruitment of smokers

From May 2019 until May 2020, participating PNs are requested to inquire about the smoking status of all their patients with smoking-related complaints. Patients who report to be a smoker (no set minimum requirements) will be asked to participate in the study. If they agree to participate, they will receive brief guidance and referral advice dependent on the condition in which the PN has been assigned. Written informed consent will be obtained from all participants. Smokers will be rewarded a gift voucher of €10 for participating if they answer both questionnaires.

Inclusion criteria will be that patients smoke tobacco products, are at least 18 years old, and able to read and understand Dutch. Patients who only use e-cigarettes/e-cigars are not eligible to participate.

### Intervention: the referral aid

The referral aid is named ‘StopWijzer’, which can be translated as either stop guide or stop wiser. The content of the referral aid is based on a needs assessment consisting of a literature review (e.g., Stanczyk et al., 2014; De Vries, 2017; de Ruijter *et al.*, 2018), individual semi-structured interviews among GPs (*n* = 5), PNs (*n* = 20), and smokers (*n* = 9), a Delphi study on the referral to EBSCIs (not published yet) and the input of an advisory board consisting of experts representing various Dutch smoking cessation-related organizations of whom six were actively involved

The PNs in the experimental condition will receive an intervention manual to aid them in discussing smoking cessation with their patients and to help them select an EBSCI that fits the patient’s needs and preferences. Smoking cessation interventions that are included in the referral aid are: (1) face-to-face counseling (Pieterse *et al.*, 2001); (2) counseling via the Internet (eHealth) (Te Poel *et al.*, 2009; Stanczyk *et al.*, 2014); (3) telephone counseling (Berndt *et al.*, 2016); (4) group counseling (McEwen *et al.*, 2006); (5) pharmacotherapy; and (6) nicotine replacement therapy. It is strongly recommended to only offer pharmacotherapy and nicotine replacement therapy in combination with a form of behavioral counseling, as it is also counseled by the Dutch smoking cessation guidelines (Chavannes *et al.*, 2017).

The use of nonevidence-based methods such as acupuncture and the use of e-cigarettes are also discussed in the referral aid as smokers might inquire about the effectiveness of these methods. The referral aid actively encourages PNs to not recommend these methods and stimulates them to advance the aforementioned EBSCIs as suitable alternatives.

**Figure 2.**
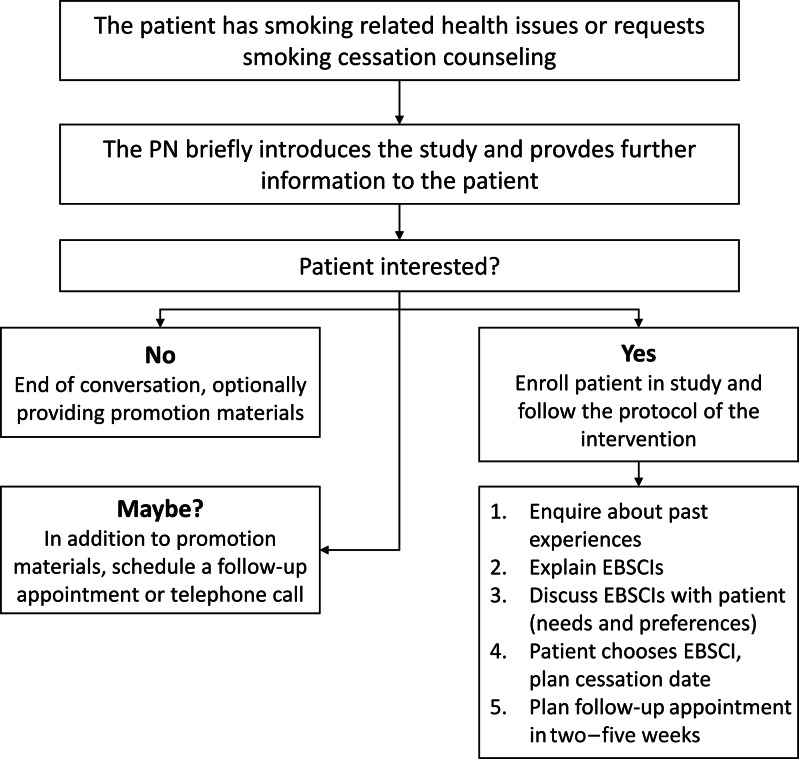
Flowchart referral aid

The PN starts with inquiring about the patients’ smoking habits and his or her interest in undertaking a smoking cessation attempt. They also inform the patient about the referral aid and the underlying study (see also Figure 2).

Patients who agree to participate in the study will be counseled in accordance with the referral aid. First, the PN inquires about earlier cessation attempts and smoking cessation methods patients may have used during these attempts. Second, the PN informs the patient about the available EBSCIs and their advantages and disadvantages based on the information provided in the intervention’s manual. If necessary, the manual also allows PNs to provide information on possible reimbursements by health insurers. Third, the PN and patient discuss which EBSCI best fits the patient’s needs and preferences. Fourth, the smoker selects an EBSCI and chooses a cessation date. Lastly, depending on the EBSCI chosen by the patient, PNs schedule at least one follow-up appointment in two–five weeks, in order to evaluate and, if necessary, to select another EBSCI.

If the smoker is not (yet) interested in participating, PNs are advised to give the smoker a flyer to take home. This flyer contains information about participating in the study and a summary of the different EBSCIs. This way, smokers may be stimulated to consider smoking cessation at a later point in time. If the smoker is uncertain about participating, PNs are advised to, in addition to handing out the aforementioned flyer, schedule a follow-up meeting or telephone call with the smoker for further discussion on participation in the study.

### Intervention materials

**Figure 3.**
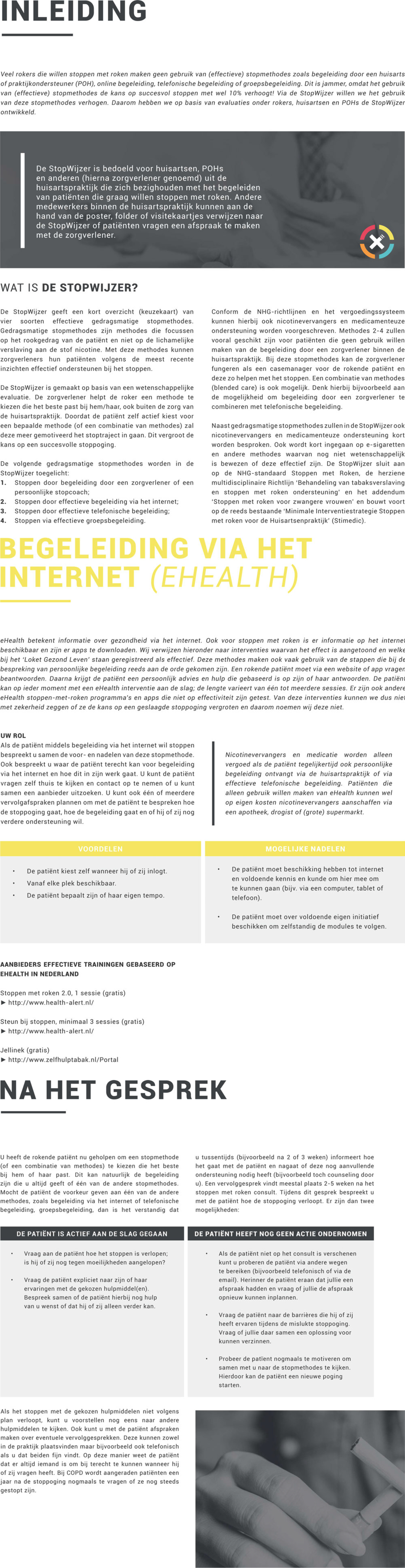
Brief overview of content interventions’ manual

Materials will consist of a small (letterbox-sized) package, which will be sent via post and a website (www.stopwijzer.nu). Taking into account the potential of low health literacy of patients, the materials have been written in a clear and comprehensible language in accordance with the applicable Dutch guidelines (Language level B1) (Ministerie van Algemene Zaken, 2019). The main component is an instruction manual for using the referral aid. This manual consists of the following elements (see also Figure 3):

1. an introduction, which explains the goals and relevance of the intervention and gives a brief overview of the different EBSCIs and the other elements of the referral aid;
2. instructions in using the referral aid, which include a roadmap of the most important steps and a summary in the form of a flowchart;
3. an overview of possible reimbursements of EBSCI’s by health insurers with a calculation tool to help patients provide insight into how much money they can save by quitting smoking;
4. an overview of the different EBSCIs, in the following order: face-to-face counseling, eHealth, counseling via telephone, group counseling, nicotine replacement therapy, pharmacotherapy, and nonevidence-based ‘cessation’ methods (acupuncture (White *et al.*, 2014), laser therapy (Kerr *et al.*, 2008), auriculotherapy (Bier *et al.*, 2002), hypnoses (Carmody *et al.*, 2008), and e-cigarettes (Kalkhoran and Glantz, 2016);
5. guidelines for following up the initial consultation;
6. some concluding remarks and room for taking notes.

#### Flowchart handout

**Figure 4.**
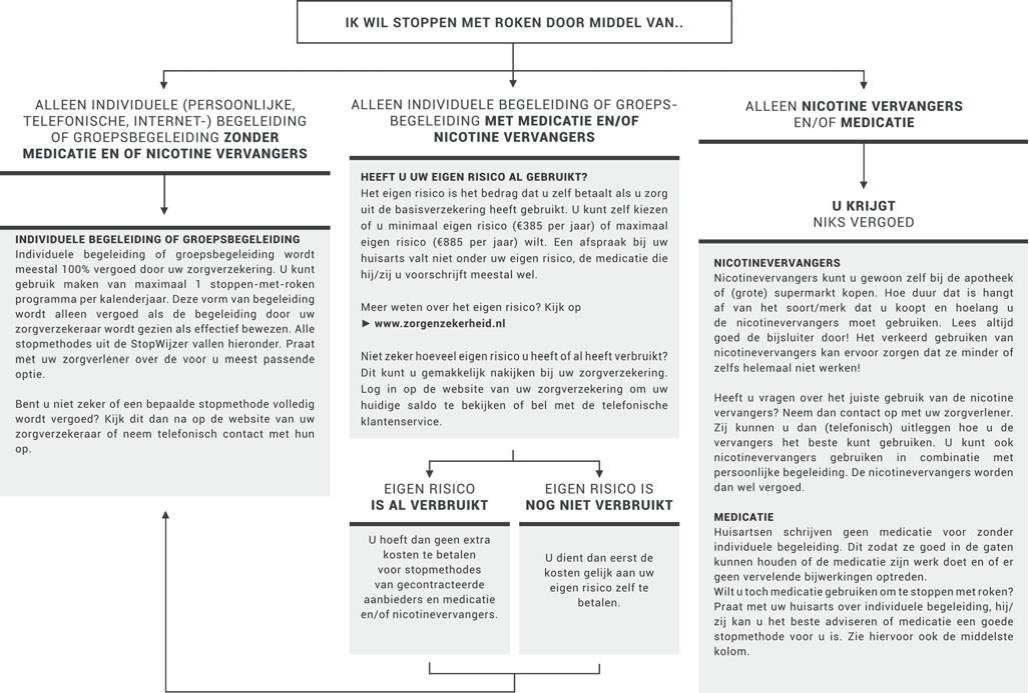
Flowchart handout

Two of the elements of the instruction manual, a summary of the referral aid and a summary of health insurer’s reimbursements policies, are printed on A5 carton handouts in order to be used by PNs as a quick reminder (see also Figure 4).

#### Placemat

**Figure 5.**
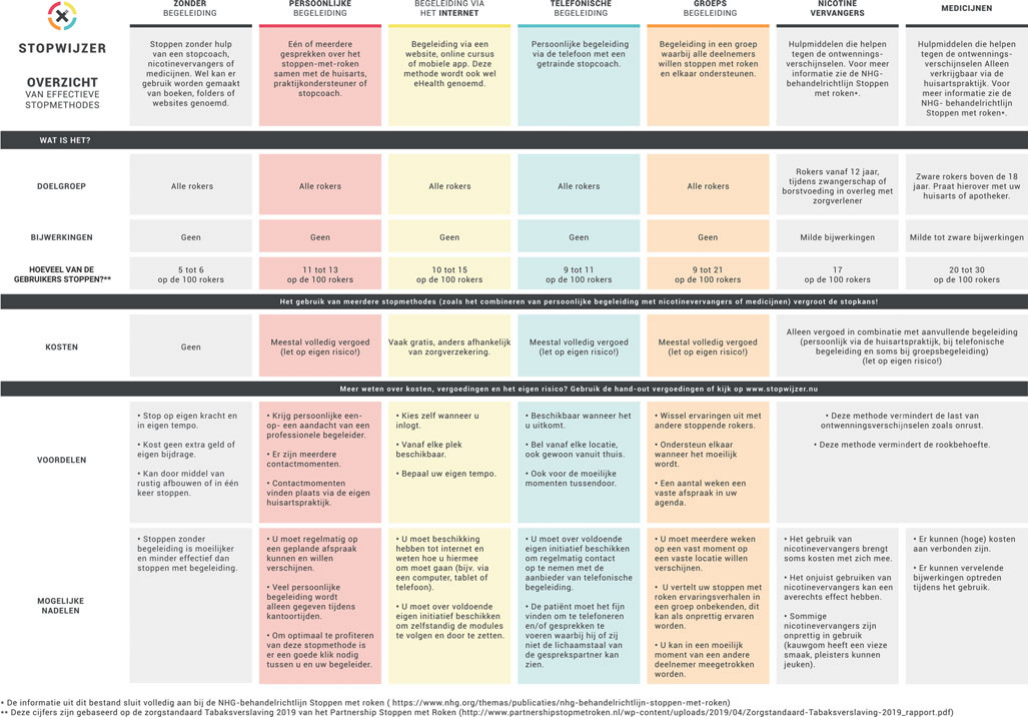
Decision matrix, provided in the form of a desk pad and available online

PNs also receive a decision matrix printed on a plastic desk pad. The desk pad can be used as a reminder for the PN or as a conversation tool during a consultation with a smoking patient (see also Figure 5). The matrix lists the EBSCIs mentioned above and gives an outline of their target groups, strengths, and weaknesses, effectiveness, and costs. The matrix can also be accessed via the project’s website.

#### Flyers

PNs will receive flyers describing 1) a summary of information on EBSCIs, 2) a list of the contact information of the research team and 3) a link to the project’s website.

#### Promotion materials

In addition, the package also will contain a stack of business cards with the contact information of the research team and a link to the project’s website. Also enclosed will be a poster, which is used to promote the project in the practice’s waiting room. A digital version of the poster that can be displayed on waiting room screens will be sent via email. Lastly, a pen and a notebook featuring the project’s logo will be included as a small reminder.

#### Website

The project’s website will consist of a general part and a password-protected part. PNs from both the experimental and the control conditions will be able to register new patients in the password-protected part. PNs from the experimental condition can furthermore access digital copies of the manual and other materials (e.g., the placemat, handouts, and flyer). Patients from the experimental condition can access materials delivering the same information as they receive from their PN. PNs and patients from the experimental condition can access a frequently asked questions (FAQ) page tailored to their needs. Smokers from the control condition can only access the general part of the website, which will contain general information about the project and the study

### Prompts to promote intervention use and to prevent questionnaire attrition

PNs will be sent a newsletter once a month in order to keep them informed of the progress of the study and to remind them of their participation. PNs who lag in recruitment will be approached personally via telephone call or practice visit. Further, PNs will be sent personal postcards during the holiday season or when they have recruited their fifth participant. Participants will be able to contact the research team via the project’s website or via email or telephone.

Additionally, to the questionnaires we sent directly to the patients, we also sent out paper questionnaires to the general practices, to be delivered to patients by the PN, which intend to reduce attrition in the period between the initial meeting and receiving the questionnaire via post or email. Smokers can complete these paper questionnaires directly after their meeting with a PN, for example, in the practice’s waiting room. Pre-addressed envelopes including postage are provided with all paper questionnaires.

### Usability testing

A prototype version of the intervention was tested by five HCPs in order to identify any ambiguities within the intervention. Open interviews were held in which the HCPs reviewed the materials together with the primary researcher and the HCPs’ comments and opinions were used as input for the final intervention. Most changes were minor and related to terminology, for example, using the term HCP instead of PN in materials to not exclude other possible participants. Other comments focused more on design elements such as making a more distinct distinction between EBSCIs and nonevidence-based interventions using visual techniques.

## Data collection

### Measures

#### Tobacco abstinence

The primary outcome of the study will be 7-day point prevalence abstinence (PPA) measured at 6-month follow-up from baseline (Mudde *et al.*, 2006; Cheung *et al.*, 2017a). Secondary outcomes are 24-hour PPA and 3-month prolonged abstinence (Mudde *et al.*, 2006). Assessing 6-month prolonged abstinence is not possible due to the study design, since follow-up measurement will take place six months after answering the baseline questionnaire irrespective of the date the participant quit smoking. Prolonged abstinence will be assessed by asking patients whether they have refrained from smoking tobacco since their last quit attempt allowing for a two-week grace period during which the participant could smoke one–five cigarettes. Furthermore, patients will be asked whether and how often they have tried to quit smoking (i.e., no cigarette for 24 h) during the intervention period. If patients indicate to smoke at follow-up, they will also be asked how many cigarettes on average they smoke per day. Because of minimal personal contact with the research staff and reduced possible contact with HCPs because of COVID-19, biochemical validation will not be possible. Therefore, all tobacco abstinence measures will be assessed at 6-month follow-up using self-report with an added ‘bogus pipeline’ question (‘Do you object if we come to do a saliva test to check your smoking status?) to reduce socially desirable responses by including the threat of biochemical testing (Aguinis *et al.*, 1993, Adams *et al.*, 2008). Previous studies suggest that the difference between self-reported abstinence rates and those verified with biochemical validation is negligible (Glasgow *et al.*, 1993; Patrick *et al.*, 1994; Velicer and Prochaska, 2004). The number of cigarettes smoked on average per day will be measured at baseline as well.

#### Smoking cessation method chosen

Patients will be asked which EBSCIs they have used in any previous smoking cessation attempts and to grade the methods they have used on a scale ranging from 1 = very bad to 10 = very good. The following EBSCIs constitute the response options: face-to-face counseling, eHealth, telephone counseling, group counseling, pharmacotherapy, and nicotine replacement therapy. If a participant uses a smoking cessation method that is not included in the response options, it will be possible to indicate this by entering a free text. The smoking cessation method selected will be assessed at a 6-month follow-up.

#### Quality of life and healthcare costs

Quality of life measures EuroQol (Janssen *et al.*, 2013) and ICECAP (Al-Janabi *et al.*, 2012) will be used to measure the incremental costs per quality-adjusted life year (QALY). Healthcare costs (e.g., productivity losses, medical consumption, and consumption of informal care) will be measured via the iMTA Medical Consumption Questionnaire [iMCQ (Bouwmans *et al.*, 2015]. The valuation of costs will be based on the latest Dutch standards, which include, for example, hourly wage of HCPs and standardized costs for consults in the GP setting (Tan *et al.*, 2012; Hakkaart-van Roijen *et al.*, 2015). Quality of life and healthcare costs will be assessed both at baseline and at 6-month follow-up.

#### Informed decision-making

Decisional conflict (e.g., ‘I feel I have made an informed choice’) will be assessed via 16 items on a 5-point Likert scale (1 = strongly disagree; 5 = strongly agree) (O’Connor, 1995; 2010). Decisional conflict will be assessed at a 6-month follow-up.

#### Contact between PN and smoker

The contact between the PN and the smoker will be evaluated because a constructive and empathic relationship between PN and smoker is an important factor for intervention success (Rogers, 2003). First, patients have to indicate which topics have been discussed during the guidance and referral advice (e.g., ‘He/she (the PN) has asked you how motivated you are to stop smoking’); (0 = yes; 1 = no). Second, the relationship between smoker and PN will be assessed via six items (e.g., During a conversation about quitting smoking, I have the feeling that my caregiver is offering me choices’) on a 5-point Likert scale (1 = strongly disagree; 5 = strongly agree). The contact between the PN and the smoker will be assessed both at baseline and at 6-month follow-up.

#### Appreciation of the intervention

After assessing which intervention materials have been noticed by the patients (e.g., ‘Have you seen the intervention’s poster in the waiting room of your general practice?’); (0 = yes; 1 = no), appreciation of the materials (e.g., ‘I think the StopWijzer materials are understandable’) will be assessed via four 5-point Likert items (1 = strongly disagree; 5 = strongly agree). Moreover, patients will be asked to grade the intervention materials (1 = very bad; 10 = very good) and patients and for comments and suggestions entering free text. The appreciation of the intervention will be assessed at a 6-month follow-up.

#### Demographics, smoking characteristics, health status, and health literacy

Demographics will be assessed via age, gender (0 = male; 1 = female), nationality (0 = other nationality; 1 = Dutch nationality), education level (1 = low: no education, primary, or basic vocational school; 2 = medium: secondary vocational school or high school; 3 = high: higher vocational school or university), and occupation of the principal wage earner of the household.

Motivation to quit smoking (e.g., Bolman *et al.*, 2002; Mudde *et al.*, 2006) will be assessed via four items. Three items (e.g., ‘I am planning to quit smoking’) use 7-point Likert scales (1 = certainly not;7 = certainly yes). One item assesses whether smokers want to quit smoking including the time frame (1 = yes, within one month; 2 = yes, within three months; 3 = yes, within six months; 4 = yes, within one year; 5 = yes, but not within one year; and 6 = no, I do not plan to quit smoking).

The intention to use a specific smoking cessation method will be assessed via 20 items (e.g., ‘In order to stop smoking, I can best make use of nicotine replacement therapy’). All items use be 7-point Likert items (1 = certainly not to 7 = certainly yes). The questions were developed for this study based on the I-Change model, which aims at explaining motivational and behavioral change via integrating various social cognitive theories (De Vries, 2017).

The current use of e-cigarettes will be assessed via one item (‘Do you use e-cigarettes?’; 1 = no; 2 = yes, without nicotine; 3 = yes, with nicotine).

Cigarette dependence will be assessed via the Fagerström Test for Cigarette Dependence (Heatherton *et al.*, 1991; Fagerström, 2011). The six items of the scale will be converted into an overall score ranging from 0 to 10. The dependence level is classified as 0–2 = low; 3–4 = moderate; 5–6 = strong; and 7–10 = very strong.

The health status of the smoker (e.g., ‘Do you have type 2 diabetes?’) will be assessed for six diseases (0=‘yes’; 1=‘no’): COPD, cancer, type 2 diabetes, cardiovascular diseases, asthma, and depression (Bouwmans, 2018).

Health literacy (e.g., ‘How often do you get help with reading letters or folders of your GP, the hospital or other health care services?’) will be assessed via three items (1 = never; 5 = always) (Chew *et al.*, 2004). All variables described in this paragraph will be assessed at baseline.

### Sample size

A power analysis for logistic regression was conducted using G * Power version 3.1 (Faul *et al.*, 2007). Considering an effect size (odds ratio) of 0.30, a power of .80, and an alpha of .05, a total sample size of 292 patients will be required. The effect size was calculated for a 10% difference between the control condition and experimental condition. Seven-day PPA was estimated to be 5% in the control condition and 15% in the experimental condition.

Assuming that patients are nested within general practices with an average cluster size of five patients, a total sample size of 300 patients will be required. Based on earlier smoking cessation studies in general practices, intra-cluster correlation (ICC) was set at .01. We will aim to recruit 60 PNs that need to recruit an average of 10 patients each. Considering a dropout rate of 50%, there will be 5 patients per GP practice that filled out the baseline questionnaire, totaling 300 patients. In order to account for drop out at six months follow-up, multiple imputations will be conducted by applying Multivariate Imputation via Chained Equations (MICE) in R (Buuren and Groothuis-Oudshoorn, 2010; Blankers *et al.*, 2015).

### Data analysis

All analyses will be performed following the intention-to-treat principle (Schulz *et al.*, 2010). To account for missing observations in the 6-month follow-up questionnaire, multiple imputations will be conducted by applying MICE in R (Buuren and Groothuis-Oudshoorn, 2010; Blankers *et al.*, 2015).

First, descriptive analyses will be conducted to describe the sample characteristics. Second, logistic regression will be used to analyze attrition, including baseline factors and conditions as predictors. Third, if sample size allows, multilevel logistic regression analyses will be performed to assess differences between conditions in 7-day PPA, 24-hour PPA, and 3-month prolonged abstinence. Fourth, analyses of variance will be performed to test for differences in decisional conflict and appreciation of the intervention materials between conditions.

The economic evaluation will involve the performance of a combination of a cost-effectiveness analysis (CEA) and a cost–utility analysis (CUA) of data collected during the baseline and 6-month follow-up measurement (see quality of life and healthcare costs). In a CEA, the effects are presented in clinical outcomes (here additional quitters). In the CEA, the incremental cost-effectiveness ratio (ICER) will be expressed as the incremental costs per additional quitter (measured as six months PPA). The primary outcomes measure for the CUA will be QALYs, measured via EuroQol (Janssen *et al.*, 2013) and ICECAP (Al-Janabi *et al.*, 2012). The economic evaluation will be performed from a healthcare and societal perspective implying that all relevant costs and outcomes will be considered. Intervention costs, healthcare costs, patient, and family costs (in a subsample), and costs outside the healthcare sector will be assessed.

## Discussion

### Potential strengths of the study

The first potential strength of the study is the use of a random allocation of PNs via a computer algorithm to mitigate possible biases within general practice settings as PNs working within the same general practice setting, but allocated to different conditions may have an impact on the implementation of the study. Second, the intervention was pilot tested among a group of potential users, both PNs and smokers, and experts from the field to test the usability and to remove ambiguities. Third, the GP setting was used as a gateway to reach the target group as most smokers visit their general practice yearly (Boerdam and Bevolkingstrends, 2016) and have a high level of trust in their GP (Guassora and Gannik, 2010), which makes it more accessible than specialty centers at the hospital level. Fourth, the intervention consists of a summary of various already proven effective smoking cessation methods, which are often underused at the moment. This study may help to increase their uptake. Fifth, a cost-effectiveness study will be performed, which will provide an estimation of the additional costs and benefits of the intervention as compared to care as usual. Sixth, in order to take into account, the potential of low health literacy of patients, the materials have been written in a clear and comprehensible language in accordance with the applicable Dutch guidelines. Lastly, using the intervention takes hardly any additional time, making it a perfect fit in the timeslots of their usual consultation sessions. This makes it easier for PNs to participate in the study.

### Potential limitations

First, PNs provided less smokers than expected, leading to a longer inclusion period and omission of also a 12-month follow-up as stated in our trial register, (NL7020/NTR7218). Yet, a 6-month study follow-up is still an acceptable period for assessing treatment effectiveness (West *et al.*, 2005).

Second, although we aim to include all eligible smoking patients who visit the participating general practices, there is a risk of selection bias by PNs. PNs may tend to invite more smokers who have already shown a willingness to stop smoking or who are deemed to be more easily motivated to participate, as also seen in other studies (Bundy, 2004; de Ruijter *et al.*, 2017a; 2017b). The conversational guidelines found in the intervention’s manual and flowchart handout are aimed to induce PNs to include all types of smokers.

Third, because smokers often just have one face-to-face meeting with a PN and we use mostly online questionnaires at the six months mark, we may face a high attrition rate. This is usual for studies that use online questionnaires (Eysenbach, 2005; Shahab and McEwen, 2009; Webb, 2009). We tried to overcome this by sending additional questionnaire packages so that patients could answer the questionnaire on-site if desired.

In order to try to prevent attrition at the follow-up assessment, we will also provide a shortened questionnaire including three of the aforementioned questions on abstinence (7-day PPA, 24-hour PPA, and 3-month prolonged abstinence) and one question asking the patients about EBSCIs used during their cessation attempt. This shortened questionnaire can be administered via email or telephone.

Lastly, the efficacy of counseling treatment is also dependent on contextual factors such as the patient–counselor relationship (Wampold, 2015). However, due to the nature of this study, targeting the use of evidence-based smoking cessation methods, assessing these contextual factors was beyond the scope of this study.

## Conclusion

This paper describes the study design for the development and evaluation of an information and decision tool to support PNs in the guidance of smoking patients and the referral to EBSCIs. The results of this study aim to provide insight into the (cost) effectiveness of an intervention aimed at promoting the use of more evidence-based smoking strategies, arriving at a more personalized referral decision, and the best way to communicate them to smoking patients. The behavioral effectiveness, as well as the cost-effectiveness, will be reported on in later papers.
